# Erratic electricity supply (*Dumsor*) and anxiety disorders among university students in Ghana: a cross sectional study

**DOI:** 10.1186/s13033-016-0053-y

**Published:** 2016-03-02

**Authors:** Abdallah Ibrahim, Genevieve Cecilia Aryeetey, Emmanuel Asampong, Duah Dwomoh, Justice Nonvignon

**Affiliations:** Department of Health Policy, Planning and Management, School of Public Health, University of Ghana, Accra, Ghana; Department of Social and Behavioural Sciences, School of Public Health, University of Ghana, Accra, Ghana; Department of Biostatistics, School of Public Health, University of Ghana, Accra, Ghana

**Keywords:** Generalized anxiety disorder, Electricity crisis, Mental disorders, University of Ghana

## Abstract

**Background:**

Ghana is currently experiencing electricity supply crisis that is believed to have some impact on the mental wellbeing of the population, especially among university students that have become increasingly dependent on uninterrupted electricity supply to fully function academically. There is no known study that explores the link between infrequent electricity supply and generalized anxiety disorders in sub-Saharan Africa. This study aimed to explore that link and determine the proportion of university students whose anxiety levels may be influenced by the electricity supply crisis that the country is experiencing at the moment.

**Methods:**

This exploratory study used the Generalized Anxiety Disorder 7-item scale (GAD-7) to conduct the study on the University of Ghana campus. The likelihood ratio (LR) test and Fisher’s Exact tests were used to determine any association between the electricity supply crisis and anxiety levels among students. Unadjusted odds ratio and corresponding confidence intervals were estimated and ordinal logistic regression technique was used for the effect of covariates on anxiety.

**Results:**

Overall, nearly 26 % of students interviewed felt nervous, anxious or on edge almost every day due to the erratic power supply. The proportion of students determined to be classified having minimal, mild, moderate and severe anxiety due to the erratic power supply was 24.2, 30.7, 22.1 and 23.1 % respectively. Students were significantly more likely to be anxious if the frequency of power outage increased (OR 1.36; CI 1.23–1.49).

**Conclusions:**

Our finding in this study suggests that although the erratic power supply does not allude to any clinical confirmation of the students having anxiety disorders, it does point to a fact that even in a resource-poor country like Ghana, where constant supply of electricity is not always guaranteed, students may not be entirely immune to the health and well-being implications of failures in some sectors of the economy such as power supply.

## Background

The magnitude of recent electricity supply crisis in Ghana is not unprecedented. However, the frequency of the current power cuts in an environment of a growing middle class, including university students—that have become accustomed to modern amenities which rely on regular electricity supply—is believed to have had an effect on the mental health of Ghanaians in the immediate and long-term [1].

The World Health Organization estimates that globally about 25 percent of a country’s population will suffer from a mental disorder in their lifetime, and at any point in time about 10 percent suffers from a psychiatric condition of which an estimated three percent in Ghana is believed to have severe form of mental disorders at any point in time [2, 3].

In Ghana, it is estimated that about 18 psychiatrists and about 1200 mental health nurses are known to be actively practicing for the estimated 2.5 million Ghanaians who suffer with at least one mental disorder [4]. The shortage of practicing psychiatrists creates a significant gap of about 98 % with regards to accessing mental health care in the country [3, 5]. Despite the staggering estimates of mental disorders in Ghana and for that matter Africa, little emphasis has been put on the country and continent’s public mental health care system.

Many factors are responsible for people experiencing mental disorders in their lifetime. These include physiological, personal history or lived experiences and environmental factors [6, 7]. The latter factor is usually a result of a confluence of many social events in the lives of individuals with mental disorders. These social events may either be influenced by individual, institutional or national measures that could lead to an immediate or remote trigger of mental distress [8].

Ghana is currently experiencing electricity supply crisis, particularly in the highly populated and urban areas, including the Greater Accra Region where many institutions of higher learning are located. The electricity supply crisis has had some impact on the socio-economic wellbeing of people, including students at institutions of higher learning such as the University of Ghana. Students in this university are dependent on the regular electricity supply to carry on with their normal academic and other lifestyle activities. Therefore, the infrequent supply of electricity to the university community would undoubtedly affect the students’ lifestyle, including their mental health and wellbeing. College students are normally confronted with a myriad of issues including academic, social and personal that has potential to influence their mental health. These issues generally would tend to change depending on the students’ socio-demographic factors as well as their progression in age, academic experience and socioeconomic status while living on campus [9].

The electricity supply crisis in Ghana, which is a systemic issue, is believed to have influenced the health and mental wellbeing of tertiary level students even more because the student population is made up of individuals who have become so dependent on modern technology such as smart-phones, tablets, laptops and other personal computers for their routine academic activities. These gargets are themselves, as well as the internet connectivity to enable them to function, heavily dependent on electricity supply for normal functionality. Therefore, the infrequent electricity supply to the university community is likely to impact on the anxiety levels of students who require regular power supply to function effectively.

Generally, there is limited population level research not only on anxiety and stress levels but on mental health as a whole in Ghana [10]. In fact, there is an even greater dearth of scientific evidence in Ghana on mental health of university student population. The few studies available about mental health issues in Ghana have mainly focused on policy related issues, the incarcerated, homeless youth and postpartum women among other issues [11–16]. The very few known studies on mental health issues among college students discussed socio-cultural differences in suicides and depressive disorders between Ghanaian and American students [8, 17]. In fact, there is only anecdotal evidence linking the recent electricity supply crisis to an increasing level of depression or anxiety in Ghana, and there is lack of scientific evidence to support that perception in the general population or among the student population. There is therefore the need for research to assess the mental health status of the University of Ghana students within the context of the individual and systemic factors that has the potential to influence their mental health and wellbeing. Further, this study aimed to add on to the literature by exploring the proportion of students who may have experienced electricity supply crisis-related anxiety disorders and answer the research question as to whether erratic electricity supply to the university community can influence generalized anxiety disorder among University of Ghana students.

## Methods

### Research setting

The study was conducted on the University of Ghana campus. The University of Ghana is the oldest and largest public university in the country. The university was founded as the University College of the then Gold Coast in 1948 for the purpose of providing and promoting university education, learning and research in the British colony. The university currently has a student population of 29,754, which constitutes undergraduate and graduate students. The undergraduate students constitute about 88 % of the total student population and the male to female population is 2:1.

### Measures used

This stratified cross sectional study used the generalized anxiety disorder 7-item scale (GAD-7) developed by Spitzer et al. [18] as a brief measure to assess generalized anxiety disorder (GAD) in the general population and adapted to measure anxiety in students influenced by erratic electricity supply [18]. The GAD-7 tool was chosen and adapted for this study because of its high rate of sensitivity (89 %) and specificity (82 %). The reliability coefficient (Cronbach’s alpha) for the scale (GAD-7) used in measuring anxiety was found to be 85.9 % indicating its appropriateness for use within the Ghanaian context.

Scores from the self-administered questionnaire, when added together, is categorized as follows: 0–4 = minimal; 5–9 = mild; 10–14 = moderate; and 15–21 = severe anxiety disorder. The socio-demographic variables measured in the study included age, sex, marital status, education level and religion.

### Sample size and sampling procedure

The sampled population consisted of the University of Ghana students who are residents within the campus of the university. All students were recruited directly from their respective hall (dormitory) of residence during the 2014/2015 academic year when the erratic electricity supply was more severe. The minimum sample size needed for the study was approximately 578 based on the following assumption:

1 $$Minimum \,sample \,size \,n = Design \,effect \times \frac{{\left( {Z_{{\frac{\alpha }{2}}} } \right)^{2} P(1 - P)}}{{e^{2} }}$$

$Z_{{\frac{\alpha }{2}}}$ is the standard normal variate (at 5 % type I error, *p* < 0.05, $Z_{{\frac{\alpha }{2}}}$ is 1.96).

*P* is the expected proportion of the population with anxiety disorder. Since this is unknown, the default proportion of 0.5 was used. The margin of error ‘*e*’ is 5 % with design effect of 1.5. By substituting the above parameters in Eq. (1), the minimum sample size obtained is approximately 578. Bearing in mind a non-response rate of 10 %, the final sample size used for the study was 650 participants from all of the 13 different halls of residence. Of the 650 students from each hall of residence, 50 students were systematically selected from each hall of residence to represent the study participants.

### Data collection procedure

The study instruments were administered to the participants in their rooms by experienced research assistants who were further trained for the purposes of this study. These research assistants first explained to the participants the purpose and the importance of their participation in this study. An average of 40 min was used for each interview. The entire data collection activity lasted for a period of 2 weeks. Participants did not receive any reward for their participation in the study.

### Statistical analysis

The likelihood ratio (LR) test and fisher’s exact test with their corresponding p values were used to determine associations between each categorical predictor and level of anxiety among students. Socio-demographic factors that were significantly associated with intensity of anxiety from the LR test were included in the multivariable analysis. Unadjusted odds ratio and corresponding confidence intervals were estimated. In assessing and quantifying the effect of covariates on the intensity of anxiety, ordinal logistic regression technique was used. Proportionality assumption for ordinal logistic regression was tested using the Brand’s test. Standard error estimation was based on bootstrapping technique. Hierarchical backward selection technique was used in determining parsimonious model. The Cronbach’s alpha was used to estimate reliability coefficient for the GAD-7 scale. Proportion of missing values was only 0.15 %. Unless otherwise stated, significant associations were observed at *p* < 0.05. Data was analyzed using Stata MP Version 13 (Stata Corporation, College Station, TX).

### Ethical considerations

Ethical clearance for this study was obtained from the University of Ghana’s ethical review committee for the humanities (ECH). The study investigators additionally obtained permission from the University of Ghana Dean of students as well as Hall Masters of all the various halls of residence for students who reside on campus.

Interviewers obtained written informed consent from each participant in the study. They were informed of their right to refuse participation in the study. Students were assured that they would not be coerced into participating in the study and that refusal to participate would not incur any reprimand since it was a voluntary study.

The questionnaires administered did not bear the names of participants or codes that could directly link them to specific responses. Interviews were conducted in the rooms of participants having indicated that they were comfortable with that. The administered questionnaires were coded and kept securely. Further, the data collected were used solely for the purpose for which it was intended.

## Results

### Characteristics of study participants

The total number of study participants was 650. All the participants were recruited from all of the 13 halls of residence located within the confines of the university. Of these, 344 (53.3 %) were males and 302 (46.7 %) were females. The mean age of the students was 21 years (SD 2.3) with the youngest and oldest participants aged 17 and 39 years respectively. Students from the humanities represented 76.8 % of the total number of students sampled for the study; Basic and applied sciences, 17.3 %; and other programs, 5.9 %. All but five students did not indicate their academic level. Of the 645 students that indicated their current academic level at the time of the study, 642 (99.5 %) were undergraduate students with 242 (37.5 %) being in their second year of study. The number of postgraduate students was three (0.5 %) and almost all of the participating students were not married 637 (98.2 %). See Table 1.

**Table 1:** Socio-demographic characteristics of study participants

Socio-demographic factors	Frequency	Percent frequency
Sex	N = 647	
Male	344	53.2
Female	303	46.8
Age in years (mean ± SD)	N = 608	
	21.0 ± 2.5	NA
Marital status	N = 649	
Single	637	98.2
Married	12	1.8
Religion	N = 649	
Christianity	610	94.0
Islamic	36	5.6
Traditional	3	0.4
Place of residence outside campus	N = 581	
Accra	416	71.6
Others	165	28.4
College	N = 643	
Basic and allied health	111	17.3
Education	2	0.3
Health sciences	36	5.6
Humanities	494	76.8
Level of education	N = 645	
First year	113	17.5
Second year	242	37.5
Third year	149	23.1
Fourth year	138	21.4
Postgraduate	3	0.5

### Anxiety disorders

Table 2 presents the distribution of generalized anxiety disorders among the students. Overall, the proportion of students that felt nervous, anxious or on edge nearly every day as a result of the erratic power supply was 25.8 %. In assessing how the impact of erratic power supply have made life unbearable on campus, 61.4 % of the students indicated that it has made their work very difficult or worse. The mean anxiety score was 9.43 (SD 5.90) and the proportion of students with minimal, mild, moderate and severe anxiety as a result of the erratic power supply was 24.2, 30.7, 22.1 and 23.1 % respectively. Female students reported a slightly higher level of moderate anxiety (23.3 %), compared to male students reporting moderate level of anxiety (21 %).

**Table 2:** Distribution of problems associated with erratic power supply from the generalized anxiety disorder 7-item (GAD-7) scale

Rate of experiencing the effect erratic power supply	Problems associated with erratic power supply n (%)						
	Feeling nervous, anxious or on edge (N = 645)	Not able to stop or control worrying (N = 643)	Worrying too much about different things (N = 644)	Trouble relaxing (N = 641)	Restless to the extent that it is hard to sit still (N = 646)	Easily annoyed or irritable (N = 646)	Feeling afraid as if something awful might happen (N = 647)
Not at all sure	174 (26.9)	205 (31.9)	158 (24.5)	126 (19.7)	184 (28.8)	211 (32.7)	313 (48.4)
Several days	196 (30.4)	174 (27.1)	220 (34.2)	195 (30.4)	178 (27.8)	174 (26.9)	144 (22.3)
Over half day	107 (16.6)	120 (18.7)	111 (17.2)	121 (18.9)	118 (18.4)	111 (17.2)	89 (13.8)
Nearly daily	168 (25.8)	144 (22.4)	155 (24.1)	199 (31.1)	150 (24.8)	150 (23.22)	101 (15.6)

n (%) represent frequency and percent frequency, N represent the number of students that responded to that particular question

### Factors associated with anxiety disorders

The availability of stand-by power generator in the hall of residence and the frequency of electricity power outage were significantly (p < 0.05) associated with level of anxiety (Table 3). The odds of reporting severe anxiety versus minimum, mild and moderate anxiety among students was slightly higher for students who experienced more than four times erratic power supply in a week compared to students who experienced less than four times erratic power supply in a week, controlling for the effect of a stand-by power generator availability in the hall of residence and programme of study (Table 4). The reliability coefficient (Cronbach’s alpha) for the scale used in measuring anxiety was found to be 85.9 %.

**Table 3:** Bivariate analysis of socio-demographic characteristics and level of anxiety based on the likelihood ratio test statistic (LR) and the corresponding p value

	Level of anxiety among students n (%)					
	Minimum anxiety (N = 152)	Mild anxiety (N = 193)	Moderate anxiety (N = 139)	Severe anxiety (N = 145)	LR	p value
Socio-demographic factors						
Sex						
Male	73 (21.9)	114 (34.1)	70 (21.0)	77 (23.1)		
Female	78 (26.7)	79 (27.1)	68 (23.3)	67 (23.0)	7.29	0.2950
Age (mean ± SD)	2.26 ± 1.43	6.94 ± 1.41	11.97 ± 1.43	17.86 ± 2.12	1.20*	0.3078**
Marital status						
Single	150 (24.3)	191 (30.9)	134 (21.7)	143 (23.14)		
Married	2 (20.0)	2 (20.0)	4 (40.0)	2 (20.0)	****	0.5820****
Religion						
Christianity	144 (24.4)	181 (30.6)	125 (21.2)	141 (23.9)		
Islamic	7 (20.6)	11 (32.4)	12 (35.3)	4 (11.8)	****	0.2900****
Traditional	1 (33.3)	1 (33.3)	1 (33.3)	0 (0.00)		
Place of residence						
Accra	95 (23.5)	128 (31.6)	86 (21.2)	96 (23.7)		
Others	33 (20.5)	52 (32.3)	38 (23.6)	38 (23.6)	0.76	0.8580
College						
BAH	29 (27.1)	37 (34.6)	21 (19.6)	20 (18.7)		
Education	2 (100)	0 (0.0)	0 (0.0)	0 (0.0)		
Health sciences	13 (37.1)	10 (28.6)	8 (22.9)	4 (11.4)	14.37	0.109
Humanities	107 (22.3)	143 (29.7)	110 (22.9)	121 (25.2)		
Level of education						
100	27 (24.5)	37 (33.9)	27 (24.8)	18 (16.5)		
200	59 (25.1)	76 (32.3)	43 (18.3)	57 (24.3)		
300	28 (19.2)	43 (29.5)	35 (24.0)	40 (27.4)	14.07	0.296
400	36 (27.1)	34 (25.6)	33 (24.8)	30 (22.6)		
Postgraduate	0 (0.0)	2 (66.7)	1 (33.3)	0 (0.0)		
Frequency of light off in a week						
1–4 times	125 (27.7)	150 (33.3)	97 (21.5)	79 (17.5)		
More than four times	22 (13.8)	37 (23.1)	40 (25.0)	61 (38.1)	35.28	<0.0001
Availability of a generator in a hall						
Yes	32 (17.2)	49 (26.3)	45 (24.2)	60 (32.3)		
No	120 (27.2)	144 (32.6)	94 (21.3)	84 (19.0)	20.27	<0.002

n (%) represent frequency and row percentages

N represent the number of students that responded to that particular question

*LR* is the likelihood ratio test statistic, *SD* standard deviation, *BAH* basic and applied health

* represents estimate from one way Anova

** p value estimate based on Kruskal Wallis test

**** represent fishers exact p value

**Table 4:** Multivariable analysis of socio-demographic factors associated with the level of intensity of anxiety disorders using ordinal logistic regression

Socio-demographic factors	Level of anxiety disorders (MAD < *MAD < **MAD < SAD)			
	Unadjusted POR (95 % CI)	p value	Adjusted POR (95 % CI)	p value
Frequency of light off in a week				
1–4 times *(ref)*	1		1	
More than four times	1.36 (1.23–1.49)	<0.0001	1.33 (1.20–1.46)	<0.0001
Availability of a generator in a hall				
No *(ref)*	1		1	
Yes	0.53 (0.39–0.73)	<0.0001	0.87 (0.63–1.20)	0.384
College				
Basic and applied health *(ref)*	1		1	
Education	****	<0.025	****	<0.012
Health sciences	0.70 (0.35–1.38)		0.66 (0.28–1.56)	
Humanities	1.41 (0.95–2.10)		1.43 (1.30–3.48)	

*ref* represent the reference category

*MAD* minimal anxiety disorder, * MAD mild anxiety disorder, **MAD moderate anxiety disorder, *SAD* severe anxiety disorder, *POR* is proportional odds ratio from the ordinal logistic regression where SAD was taken as the key outcome with reference to MAD

* MAD, and ** MAD, **** indicates that confidence interval estimates from bootstrapping were not possible

### Sources of electricity and frequency of erratic power supply

The proportion of students that used other sources of light when there was no electricity power supply was as follows: Rechargeable lamp (21.9 %), torchlight (19.5 %), cell phone (20.6 %), candle (6 %), two or more of the light sources stated above (22.5 %), other light sources different from the ones stated above (3.2 %) and no other light source whatsoever (10.8 %). Regarding the activities carried out when there is electric power cut, the distribution of the students was as follows: Go to other halls of residence that had electric power (13.9 %); go to lecture theatre (5.7 %); leave the university campus (4.3 %); use other (27.8 %); and do not involve in any activities when there is light out (36.9 %). At the time of data collection, 450 students (representing 70.0 % of the sampled students) indicated that there was no stand-by power generator in the hall of residence. Of the remaining 196 students that stated that there was a stand-by power generator in their hall of residence, 181 (92.3 %) indicated that the stand-by power generator was put to use only when the power from the electricity company of Ghana was out. Out of the 626 students that stated the number of times they experienced light off in a week, 462 (73.8 %) indicated that they experience erratic power supply one to four times in a week and 164 (26.2 %) experienced light off more than 4-times in a week.

## Discussion

This study aimed to explore the possible link between the erratic electric power supply in Ghana and anxiety levels among tertiary students. It also aimed at contributing to the literature on mental health issues among African university students by exploring the proportion of students who experience electricity supply crisis-related anxiety disorders. This study also answers the research question about how erratic electricity supply to the university community may influence generalized anxiety disorder among University of Ghana students.

Generally, it is believed that the recent electricity supply crisis in Ghana has affected the mental wellbeing of Ghanaians, including students at the tertiary level institutions. Students are believed to be particularly affected because that population is made up of a generation that have become increasingly accustomed to the use of modern technology, which in itself is highly dependent on the availability of uninterrupted power supply from the national grid.

We found in this study that overall, nearly 26 % of the students that participated in the study indicated that they felt nervous, anxious or on edge “almost daily”. This suggests that the erratic power supply in the country influenced the mental wellbeing of more than a quarter of the tertiary level students. We also found that more than 30 % indicated having the same feeling on “several days” due to the erratic power supply situation in the country. Additionally, 23.1 % of the students in this study were found to have had severe generalized anxiety disorder as a result of the erratic power supply. This finding is higher than those classified in a sub-threshold with high severity of GAD symptoms that Kanuri et al. [19] found in their study in which they evaluated anxiety screening in college students that met the diagnostic and statistical manual of mental disorders, Fifth edition (DSM-V) clinical criteria for GAD [19].

Since our findings in this study suggests a link between the erratic electricity power supply and anxiety level among university students in Ghana, it could be due to a number of factors, including students’ inability to carry on with their routine academic activities such as conducting online search, completing class assignments and overall academic work that is increasingly becoming technology dependent in Ghana. The students’ anxiety level could also be influenced by the fact that the electricity disruption impacted their ability to prepare meals. This is because some students on Ghanaian university campuses tend to prepare their own meals in their halls of residence, unlike their counterparts in places like the U.S. where students on university campuses obtain their meals already prepared by vendors [20]. Therefore, any disruption in the flow of electricity, which affects their ability to prepare meals, can be very challenging. The link between poor electric supply and students’ anxiety level found in this study corroborates other finding on drought-induced disrupted electric power supply and its effect on the overall health of individuals who are dependent on constant electricity supply for survival [6]. Therefore, regardless of what the causes are, with regards to disrupted power supply, those who rely on the consistent supply of electricity are more likely to suffer from a mental health condition as a result of any power supply disruption.

Although in this study we found that female students had a slightly higher prevalence level of moderate anxiety, compared to their male counterparts, there was no such difference in the prevalence level in the severe anxiety category when both sexes were compared. The higher prevalence of moderate level of anxiety found in female students compared to their male counterparts in our study was somewhat similar to what other studies found [21, 22].

Despite the fact that the electricity power supply company, the electricity company of Ghana (ECG), had made an attempt to publish a schedule of when and where there would be power outage for each community so that people can plan ahead or anticipate disruption, the students on the university campus were still found in this study to have experienced some amount of generalized anxiety disorder, especially among the undergraduate level students. In fact, we found that in all the undergraduate student class levels, including levels 100–400 (freshmen–seniors), there were self-reported moderate (18.3–24.8 %) and severe (16.5–27.4 %) prevalence levels of GAD.

Although our overall finding of more than a quarter of students that felt nervous, anxious or on edge nearly every day due to the disrupted electricity supply seem consistent with the World Health Organization’s estimates that of any given population, about 25 % will suffer from a mental disorder, it is important to note that our study population lived in an environment that was characterised as a crisis moment of electric power supply. As discussed earlier, several factors contribute to individuals developing either episodic or chronic mental disorders and for that matter, the electricity supply crisis was found in this study to influence the university students’ feeling of GAD. This therefore goes to suggest that in spite of efforts by the power company to minimize any impact of the infrequent power supply to the Ghanaian population through the publication of outage schedule, the university students’ level of anxiety resulting from the outages was still apparent.

This study on the mental health effect, especially on anxiety levels, influenced by erratic power supply has some local, national and global implications. First, it provides important opportunity for policy development and implementation that will address the potential for health related effects of poor electric power supply in Ghana and elsewhere in Africa. This is particularly relevant now because other African countries, including in South Africa, have recently begun electric power rationing and for that matter the health and mental wellbeing of their population will likely be affected by this systemic electric supply issue as it is in Ghana.

The findings are also very important to the university administrators who now have the scientific research information to enable them to plan and put in place measures to minimize and/or address the mental health effect of the erratic electric power supply to the student population. This we believe can be done through evidence-based informed planning to enable them to institute screening, brief intervention and referral to treatment for those that may be impacted by the poor electric power supply.

Further, our research is in line with one of the objectives of the World Health Organization’s “Mental health action plan: 2013–2020” that includes strengthening evidence and research on mental disorders, especially since there is dearth of research on mental disorders, including on students in low-middle income countries like Ghana [23].

### Study limitations

This novel study on the link between the disrupted power supply and anxiety level among students is not without some limitations. The first limitation of this study is that the GAD-7 scale used for this study does not conclusively confirm that respondents indeed has anxiety disorders since the GAD-7 provides only probable diagnoses that requires confirmation in a further evaluation by a clinician. Also, the GAD-7 measures only a specific mental disorder and for that matter, students who experienced other co-occurring mental disorders with symptoms similar to that of GAD may have been included in the overall prevalence of GAD in the student population. Additionally, since our study did not evaluate the link between students’ work load and anxiety levels, it is possible that other factors may have influenced students’ anxiety levels during the electric power outages but not necessarily the erratic power supply itself. Furthermore, the lack of information about students’ with pre-existing conditions of mental disorders such as depression or stress is a major limitation of this study.

In spite of the limitations of this study however, it is important to have conducted the study in order to provide baseline information on the prevalence of GAD among college students in Ghana.

Furthermore, additional research is needed to assess the true impact of the link between the poor power supply and anxiety levels among tertiary level students in Africa, especially since a greater understanding of this condition among tertiary level student population who are exposed to various levels of emotional stressors is needed.

## Conclusions

This exploratory study provided valuable information on the link between disrupted power supply at a national level and its impact on the health of the population, particularly for groups that are heavily reliant on constant electric power supply for their daily activities. In fact, the study underscores the point that even in resource-poor countries including Ghana, where it is often not expected for issues such as irregular electricity supply to have an impact on the mental well-being of the people, it may indeed have an influence on certain groups of the population, including students whose anxiety levels are now known to be associated with power supply crisis.
